# Ferroptosis involves in intestinal epithelial cell death in ulcerative colitis

**DOI:** 10.1038/s41419-020-2299-1

**Published:** 2020-02-03

**Authors:** Minyi Xu, Jin Tao, Yidong Yang, Siwei Tan, Huiling Liu, Jie Jiang, Fengping Zheng, Bin Wu

**Affiliations:** Department of Gastroenterology, The Third Affiliated Hospital of Sun Yat-Sen University, Guangzhou, China

**Keywords:** Molecular biology, Gastroenteritis

## Abstract

Ferroptosis has recently emerged as an iron-dependent form of nonapoptotic cell death, which is also a regulated necrosis process and a response to tumor suppression. However, whether ferroptosis is involved in ulcerative colitis (UC) is unknown. The aims of this study were to investigate whether the ferroptosis is involved in UC, particularly intestinal epithelial cell (IEC) death, and to analyze the effect of the nuclear factor kappa Bp65 subunit (NF-κBp65) on ferroptosis. The gene expression of ferroptosis-related proteins was assessed in intestinal mucosal samples from human UC. The experimental model of UC was induced with dextran sulfate sodium (DSS). Ferroptosis of IECs was evaluated, the effect of NF-κBp65 on ferroptosis was analyzed by using IEC-specific *NF-κBp65*-deleted mice (p65^IEC-KO^), and the ferroptosis signaling pathway was investigated in vitro and in vivo. The results showed that ferroptosis was significantly induced in the IECs from UC patients and mice with colitis, and the ferroptosis was mediated by endoplasmic reticulum (ER) stress signaling. The specific deletion of IEC *NF-κBp65* clearly upregulated ferroptosis and exacerbated colitis, and the result showed that phosphorylated-NF-κBp65 significantly inhibited ER stress signaling by directly binding eukaryotic initiation factor 2α. These data indicate that ferroptosis contributes to UC via ER stress-mediated IEC cell death, and that NF-κBp65 phosphorylation suppresses ER stress-mediated IEC ferroptosis to alleviate UC. The results suggest that ferroptosis involves in IEC death in UC, NF-κBp65 play a critical role in the ferroptotic inhibition, and ferroptosis is a potential therapeutic target for UC.

## Introduction

Ulcerative colitis (UC) is a chronically relapsing inflammatory disease of the colon and rectum, which is clinically characterized by abdominal pain, rectal bleeding, and diarrhea^1^. Although not fully understood, the etiology of UC is generally thought to be genetically based with contributions from gut microbial factors and a disordered immune response^2^. Recent studies have revealed the main immunological/inflammatory mechanisms of UC, and have provided new methods for modulating inflammation, but few have explored the biological changes in colonic intestinal epithelial cells (IECs).

Ferroptosis has recently emerged as a newly discovered form of regulated necrosis, and it presents features that distinguish it from other kinds of cell death at the cell morphology, biochemistry, and genetics levels^3,4^. Shrunken mitochondria; condensed dense mitochondrial membranes; and reduced mitochondrial crista are the main morphological features of ferroptosis^3,4^. Iron accumulation and increased reactive oxygen species (ROS) production from lipid peroxidation play crucial roles in triggering ferroptosis^3^. In addition, downregulation of glutathione peroxidase 4 (GPX4), a scavenger of lipid ROS and increased mRNA levels of prostaglandin-endoperoxide synthase 2 (PTGS2) are key characteristics of ferroptotic cell death^5^. Although its biological function and molecular mechanism have not been thoroughly elucidated, ferroptosis has been implicated in multiple diseases^5–8^. Interestingly, in previous studies by different groups, the administration of iron chelator was found to significantly reduce ROS in colonic tissues of UC patients and to ameliorate clinical symptoms and improve endoscopic presentations^9,10^. In contrast, high dietary iron supplements exacerbated UC symptoms in both patients and murine models^11,12^. These findings indicated the important effects of iron and ROS on the pathogenesis of UC. However, whether iron- and ROS-triggered ferroptosis involves in UC remains unclear.

Endoplasmic reticulum (ER) stress has long been considered a promoting factor in UC. Emerging evidence has shown that ER stress signaling has close cross-talk with ferroptosis. Protein kinase-like endoplasmic reticulum kinase (PERK) is a major sensor of ER stress. In response to ER stress, PERK is activated through phosphorylation, and then activates and governs the downstream signaling of the eukaryotic initiation factor 2, α subunit (eIF2α)/activating transcription factor 4 (ATF4)/C/EBP-homologous protein (CHOP) pathway^13^. PERK/eIF2α signaling was found to regulate the production of ROS during ferroptosis^14–16^. It was reported that the activation of the PERK branch facilitated whole cigarette smoke condensate-induced ferroptosis in human bronchial epithelial cells^14^. Results from our recent study demonstrated that ER stress signaling promotes colonic epithelial apoptosis in UC^17^. However, the role of ER stress-mediated ferroptosis in UC remains poorly understood and needs to be further investigated.

The transcription factor nuclear factor kappa B (NF-κB) has been confirmed to exert principal function on the modulation of UC. NF-κB family is composed of five members and the activity of NF-kB is mainly initiated by the phosphorylated subunit p65. Recently, numerous studies reported that the effects of activated NF-κB are cell type-specific. NF-κB promotes cytokine and chemokine production in inflammatory cells^18^, whereas, it also functions in IECs to maintain physiological homeostasis and contribute to damage resistance^19^. Our previous study revealed that the application of the NF-κB inhibitor BAY 11-7082 enhanced epithelial apoptosis in experimental colitis^20^, suggesting a role for activated NF-κB in inhibiting apoptosis. Moreover, growing evidence has indicated that NF-κB is involved in the regulation of ER stress signaling and ferroptosis process^21,22^. However, the definite protective mechanisms of NF-κB via the regulation of ER stress-mediated ferroptosis in UC are not completely understood.

In our present study, the effect and mechanism of ferroptosis in UC via the regulation of NF-κBp65/ER stress were investigated. We report first that ferroptosis is involved in the IEC cell death of UC that is mediated by ER stress signaling. Specifically, we found that phosphorylated-NF-κBp65 protects the IECs against ferroptosis by suppressing ER stress, suggesting that ferroptosis and NF-κBp65 phosphorylation are potential therapeutic targets for UC treatment.

## Materials and methods

### Human colonic tissue samples

Colonic mucosal specimens from patients with active UC (Mayo endoscopic score ≥ 2)^23^ and healthy controls were collected from the Gastroenterology Department of the Third Affiliated Hospital of Sun Yat-Sen University. Written informed consent was signed by each study participant. The acquisition of these samples was approved by the Clinical Research Ethics Committee of The Third Affiliated Hospital of Sun Yat-Sen University ([2018] 02-409-01).

### Microarray experiment

Three paired colonic mucosal samples from colitis patients and control individuals were collected. Total RNA from each sample was extracted and reverse transcribed into cDNA. The cDNA was then processed for gene expression microanalysis as previously described^24^. The Gene Expression Omnibus (GEO) accession number from NCBI is GSE134025.

### Animal experiments

All animal experiments in this study were conducted with the approval of the Institutional Animal Care and Use Committee at Sun Yat-Sen University. All mice involved had a C57BL/6 gene background. Wild-type mice were obtained from the Laboratory Animal Center of Guangdong Province. Mice carrying the *loxP-*flanked *NF-κBp65* allele [*NF-κBp65*^*flox/flox*^] were precious gifts from Dr. Jianping Ye [Pennington Biomedical Research Center, Louisiana State University System, Baton Rouge, LA, USA]. *Villin*-cre transgenic mice were purchased from The Jackson Laboratory. Mice with IEC-specific *NF-κBp65*-deletion [p65^IEC-KO^] were generated by breeding *NF-κBp65*^*flox/flox*^ mice with *villin-cre* transgenic mice, and the *NF-κBp65*^*flox/flox*^ littermates were used as wild-type (WT) mice. All mice were housed in rooms under controlled condition, with room temperature and with 50% humidity and 12-hour light–dark cycles. All mice with age- and sex-matched between 6 and 8 weeks of age were assigned randomly to groups. To induce experimental colitis, the mice were challenged with 3% dextran sulfate sodium (DSS; MP Biomedicals, LLC, Solon, OH) in drinking water for 7 days. The control mice were allowed to drink water only at the same time. To administer ferrostatin-1 (Fer1) in vivo, we intraperitoneal injected mice daily with Fer1 (Merck, Darmstadt, Germany, 2.5 μmol/kg body weight)^25^, and the corresponding control mice were injected intraperitoneally with normal saline. To treat mice with GSK2606414 (GSK 414) in vivo, the mice were administered either GSK 414 (Selleck, Shanghai, China, suspended in vehicle solution containing 0.5% hydoxypropylmethyl cellulose and 0.1% Tween 80 in water at pH 4.8, 50 mg/kg body weight)^26,27^ or vehicle solution by oral gavage daily during DSS administration.

### Cell culture and drug treatment

The HCoEpiC cell (human normal colonic epithelial cell) was cultured in colonic epithelial cell medium (CoEpiCM, ScienCell Research Laboratory, CA, USA) containing 10% fetal bovine serum and other supplements according to the manufacturer's instructions (ScienCell Research Laboratory). To induce ferroptosis, cells were seeded on 12-well plates and treated with RSL3 (Selleck, 20 μm) for 8 hours after plating. For the ER stress suppression experiment, 1 μm GSK 414 was added to the medium 30 minutes before RSL3 challenge. For the TNF-α treatment experiment, the cells were administrated with TNF-α (PeproTech, Rocky Hill, NJ, USA, 40 ng/ml) for 1 hour and the NF-κB inhibitor, BAY 11-7085 (Merck, 10 μm), was added to some wells 15 minutes before the TNF-α was added.

### Statistical analysis

All data were expressed as the means ± SEM. Data were statistically analyzed by SPSS 22.0. Differences in two groups were analyzed using Student’s *t* test. Comparisons of multiple groups were analyzed by one-way ANOVA analysis of variance followed by post hoc Bonferroni correction. Differences were considered statistically significant when *P* < 0.05. Unless indicated otherwise in the figure legends, all experimental results were based on *n* = 6 mice or human samples per group, and all experiments were repeated twice. Information on the additional materials and methods used in this study can be found in the Supplementary materials and methods.

## Results

### Ferroptosis was induced in UC in both human and mice

**Fig. 1:**
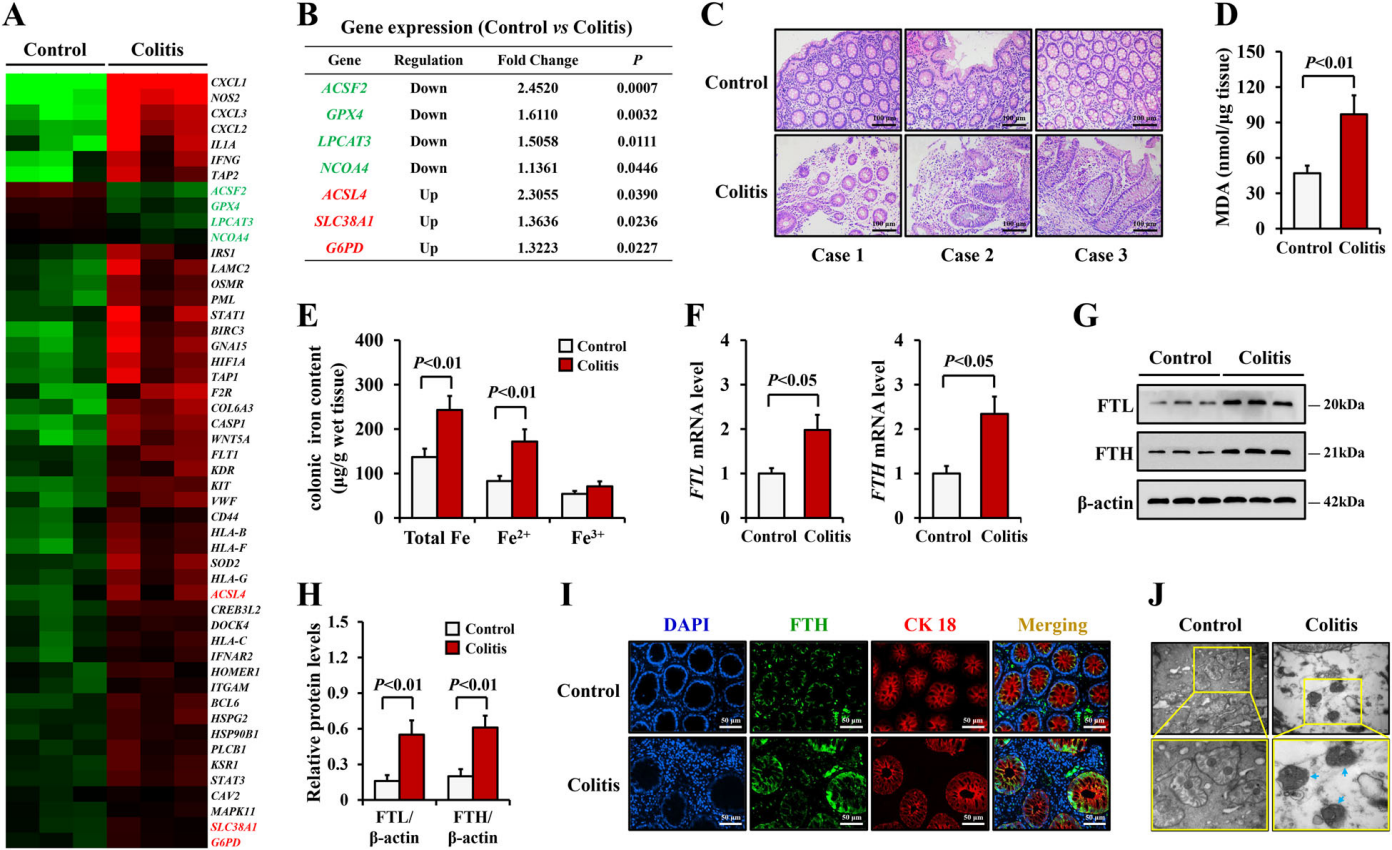
Ferroptosis was induced in colitis in human. **a**, **b** Microarray results showed ferroptosis-related genes expression in human colonic biopsy samples from control and ulcerative colitis (UC) patients (*n* = 3). The fold changes in mRNA levels in UC relative to control are represented by green and red squares, showing decreased and increased levels, respectively. The ratio represents the expression value in UC tissues compared with the expression level in control tissues. *P* < 0.05 by using Student’s *t* test. **c** Representative images from H&E staining of colon tissues from control and UC patients (Scale: 100 μm). **d** MDA levels were measured according to MDA Assay Kit. **e** Iron levels of colonic biopsy tissue were determined by Iron Assay Kit. **f** mRNA levels of FTL and FTH were detected by real-time PCR. **g**, **h** Western blotting analysis of FTL and FTH. β-actin was used as the loading control. **i** Double immunofluorescent staining for FTH and cytokeratin 18 (CK 18) were performed in the colonic sections of control and UC patients. Nuclei was stained with DAPI in blue. Localization of FTH was visualized in green and CK 18 was stained in red, the merging positive signals were visualized in yellow (Scale: 50 μm). **j** Transmission electron micrographs of colonic epithelial cells from human UC and control samples (Scale: 500 nm). Blue arrows indicate shrunken mitochondria. Statistical analyses were performed with Student’s *t* tests.

In recent decades, multiple studies have revealed contributing roles of ROS and iron in the progression of UC^28,29^. Because ROS accumulation and iron overload are critical steps in the initiation of ferroptosis, we hypothesized that ferroptosis is implicated in UC. To verify this hypothesis, we collected colonic mucosal specimens from three UC patients and three control individuals to perform a gene expression microanalysis. Among the mRNA transcriptomes revealed through this detection, dozens of genes that had been previously identified as highly expressed in UC were also elevated in our data (Fig. 1a and Supplementary table 1)^30,31^. Interestingly, several reported ferroptosis-associated genes, namely, acyl-CoA synthetase family member 2 (ACSF2), GPX4, lysophosphatidylcholine acyltransferase 3 (LPCAT3), nuclear receptor coactivator 4 (NCOA4), acyl-CoA synthetase long-chain family member 4 (ACSL4), solute carrier family 38, member 1 (SLC38A1), and glucose-6-phosphate dehydrogenase (G6PD), all of which participate in the regulation of lipid or iron metabolism^3,4,32^, were remarkably downregulated or upregulated in the UC specimens in our study (Fig. 1a, b). This gene analysis indicated that ferroptosis may be involved in colitis. In addition, the clinical UC samples presented with broken epithelium, damaged crypts, and inflammatory cell infiltrations, findings that were histologically consistent with the characteristics of UC (Fig. 1c).

To confirm that ferroptosis is involved in colitis, we then measured MDA levels and iron contents in the colonic tissues. Compared with the control samples, the colitis specimens had significantly higher MDA and iron levels, particularly ferrous iron. (Fig. 1d, e). Ferritin is a cellular iron storage protein composed of two similar polypeptide chains: ferritin light chain (FTL) and ferritin heavy chain (FTH). Existing researches have shown that increased cellular iron levels during ferroptosis induce transcriptional upregulation of ferritin^14,32,33^. Indeed, we found that both the mRNA and protein levels of FTL and FTH were obviously increased in the UC tissues (Fig. 1f–h). Immunofluorescent assay revealed that the elevated positive signals for FTH were primarily evident in the epithelial cells (Fig. 1i), suggesting that ferroptosis mainly occurred in the epithelial cells. Moreover, we observed shrunken mitochondria in the UC samples by transmission electron microscopy (Fig. 1j), a finding consistent with the morphological characteristic of ferroptosis.

**Fig. 2:**
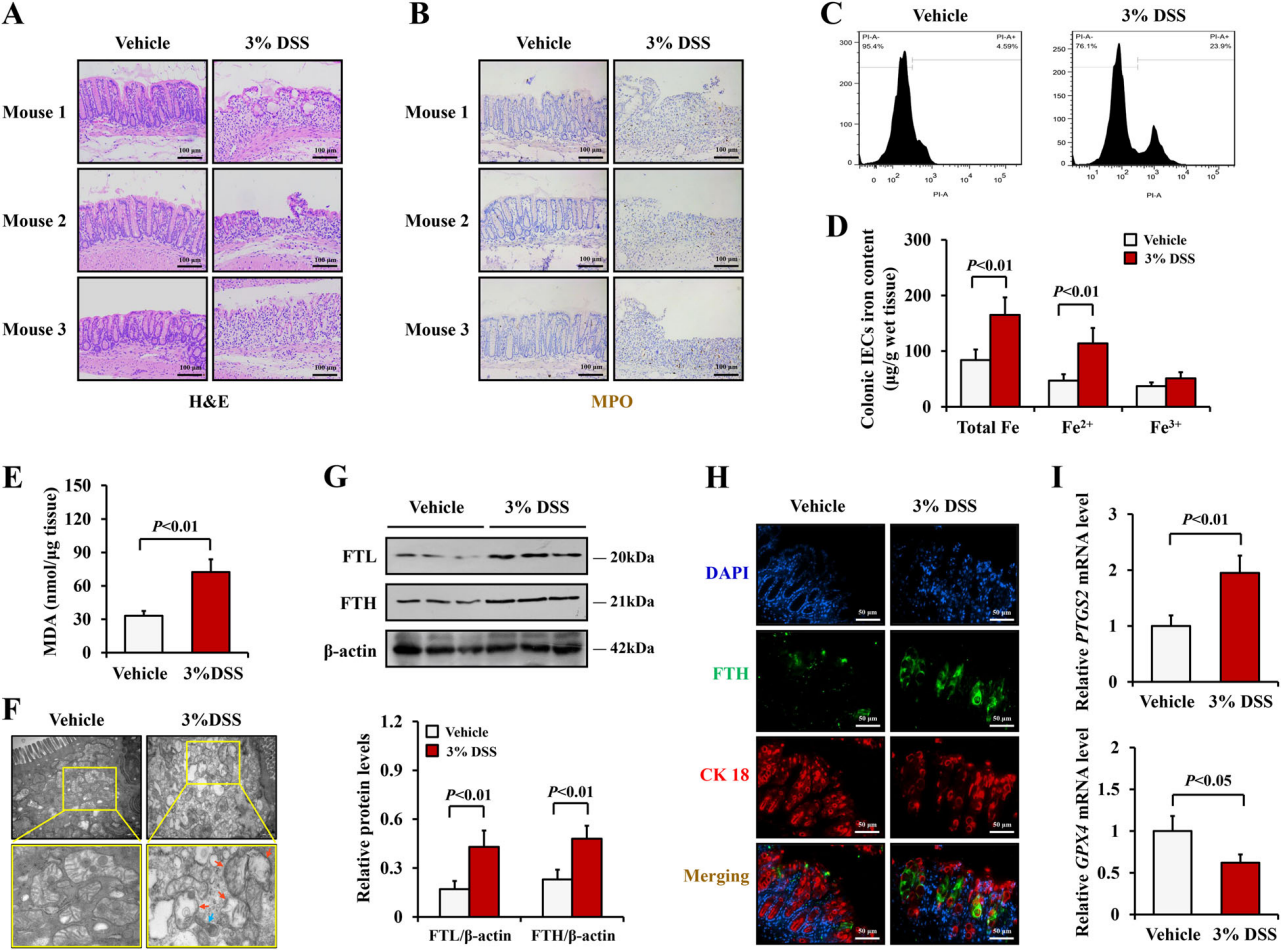
Ferroptosis was induced in colitis in mice. **a** Representative images from H&E staining of colonic sections from DSS-treated mice and vehicle mice (Scale: 100 μm). **b** Immunohistochemical staining for MPO of colonic sections (Scale: 100 μm). **c** PI-positive cells were detected by flow cytometry to analysis necrotic cell death in colonic epithelial tissues of mice. **d** Iron levels of colonic epithelial tissues of mice were determined by Iron Assay Kit. **e** MDA levels were detected in colonic epithelial tissues of mice according to MDA Assay Kit. **f** Transmission electron micrographs of colonic epithelial cells from experimental colitis and vehicle mice (Scale: 500 nm). Blue arrows indicate shrunken mitochondria and red arrows indicate reduction of mitochondria crista. **g** Western blotting analysis of FTL and FTH in colonic epithelial tissues. β-actin was used as the loading control. **h** Double immunofluorescent staining for FTH and CK 18 were performed in the colonic sections of mice. Nuclei was stained with DAPI in blue. Localization of FTH was visualized in green and CK 18 was stained in red, the merging positive signals were visualized in yellow (Scale: 50 μm). **i** Relative mRNA level of PTGS2 and GPX4 in colonic epithelial tissues from mice with or without DSS administration were measured by real-time PCR. Statistical analyses were performed with Student’s *t* tests.

In light of the results described above for human UC, we induced experimental colitis in mice by challenging them with 3% DSS for 7 days. DSS administration led to inflammatory damage in the colonic epithelium and abundant leukocytes, which were particularly evident with the neutrophil infiltration in the mucosa (Fig. 2a, b). Ferroptosis is a kind of programmed necrotic cell death, which can be identified through propidium iodide (PI) staining. Herein, we injected PI solution into mice before killed. The extracted colonic IECs were subject to flow cytometry to detect PI-positive cells. We found that the number of PI-positive IECs was increased in colitis mice (23.9% in the DSS group vs 4.59% in the vehicle group) (Fig. 2c). Consistent with the data on human UC, colonic IECs from the DSS-treated mice had shrunken mitochondria and reduced mitochondrial crista (Fig. 2f), and with elevated levels of intracellular iron and ROS as well as upregulated FTL and FTH (Fig. 2d, e, g, h). Moreover, we observed increased *PTGS2* and decreased *GPX4*, which were generally used as biomarkers of ferroptosis^3,4,32^, in the colonic IECs from experimental colitis mice, compared with the vehicle mice (Fig. 2i). These data indicated that the colonic epithelial cells of the DSS-treated mice underwent ferroptosis.

On the basis of the findings described above, we concluded that ferroptosis was induced in the colon, predominately in the colonic IECs of UC.

### Inhibition of ferroptosis alleviated experimental colitis in mice

**Fig. 3:**
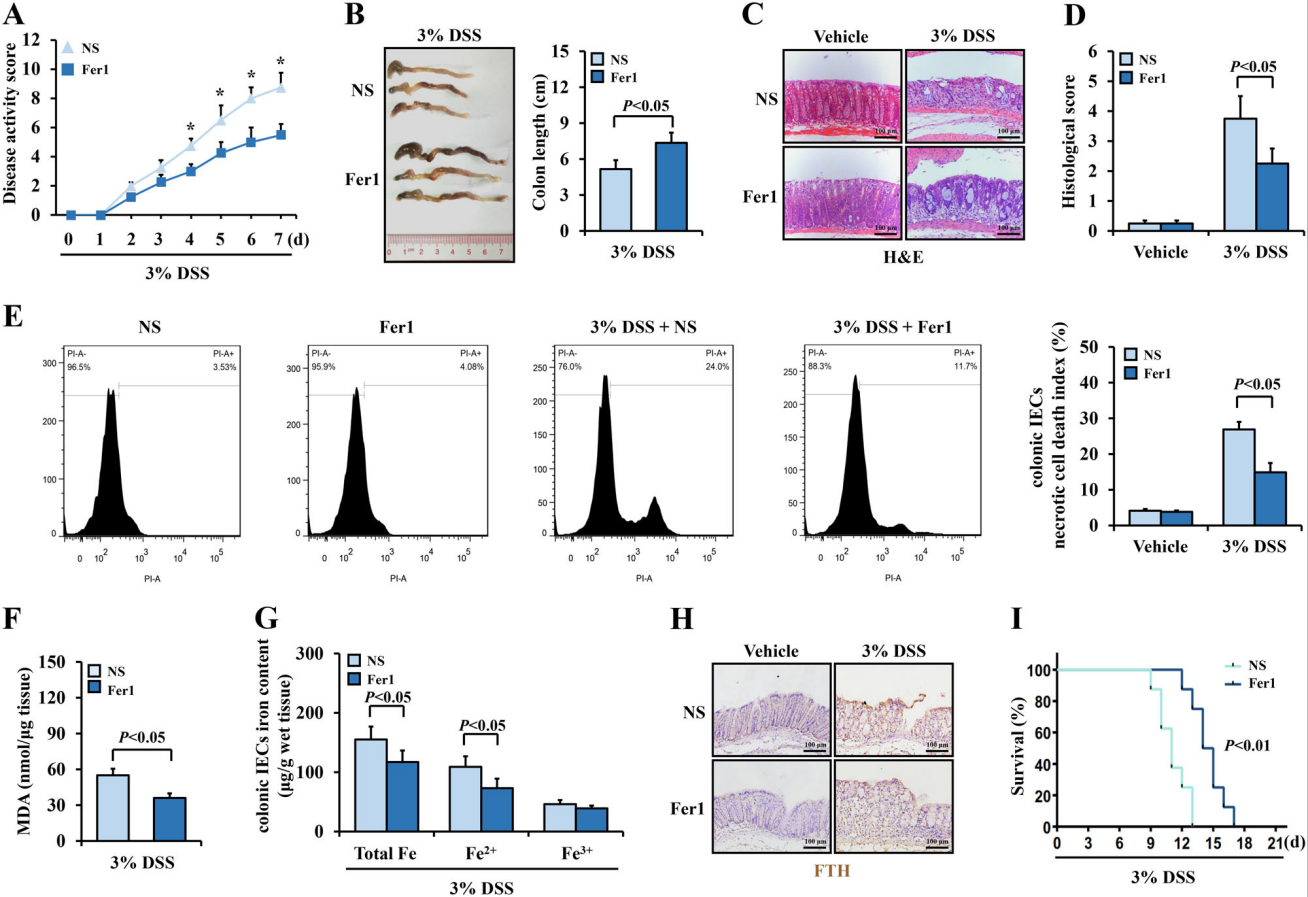
Inhibition of ferroptosis alleviated experimental colitis. Mice were treated with ferrostatin-1 (Fer1) or normal saline (NS). **a** Comparison of the disease activity index between DSS-challenged mice received Fer1 or NS treatment (*n* = 8 in each group). *P < 0.05 versus mice from NS group. **b** Comparison of colon length between colitis mice received Fer1 or NS treatment. **c** Representative images from H&E staining colonic sections from Fer1 group and NS group mice (Scale: 100 μm). **d** Histologic scores were determined according to H&E-stained sections. **e** Flow cytometric detection of PI-positive cells to analysis necrotic cell death in colonic epithelial tissues of mice. **f** MDA levels were detected in colonic epithelial tissues. **g** Iron levels of colonic epithelial tissues. **h** Immunohistochemical staining for FTH of colonic sections (Scale: 100 μm). **i** Kaplan–Meier survival curve from 3% DSS administrated mice received Fer1 or NS treatment (*n* = 8 in each group). Statistical analyses were performed with Student’s *t* tests (two groups) or one-way ANOVA (more than two groups).

Fer1 was the first identified selective small-molecule inhibitor of ferroptosis, and a large number of studies have verified its efficiency in suppressing ferroptosis both in vivo and in vitro^3,7,25,34^. To determine the role of ferroptosis in colitis, we adopted Fer1 to mice. Interestingly, Fer1 treatment significantly reduced the disease activity score and ameliorated colon length shortening in the experimental colitis mice (Fig. 3a, b). Histological analysis further confirmed the ameliorating effects of Fer1 on colitis, including lessened mucosal erosions and descended inflammatory infiltrations (Fig. 3c, d). These effects were observed after ferroptosis of colonic IECs has inhibited, as necrotic cell death in the colonic IECs from DSS-treated mice was obviously rescued by Fer1 (11.7% in the DSS + Fer1 group vs 24.0% in the DSS + NS group) (Fig. 3e). Furthermore, the initial high levels of MDA, iron contents, and FTH were decreased after Fer1 administration (Fig. 3f–h). Deferoxamine (DFO) is another ferroptosis inhibitor that chelates excessive free iron to reduce ferroptosis. Similar to the effects of Fer1, treatment with DFO also alleviated colitis and reduced necrotic cell death of the IECs (Supplementary figure 1). To investigate the survival of the mice, we subjected them to a longer period of 3% DSS treatment and Fer1 administration. The results showed that Fer1 treatment notably prolong the survival days of mice with colitis (Fig. 3i). Taken together, these results indicated that blockage of ferroptosis by Fer1 ameliorated DSS-induced murine colitis.

### Ferroptosis was mediated by ER stress in colitis

**Fig. 4:**
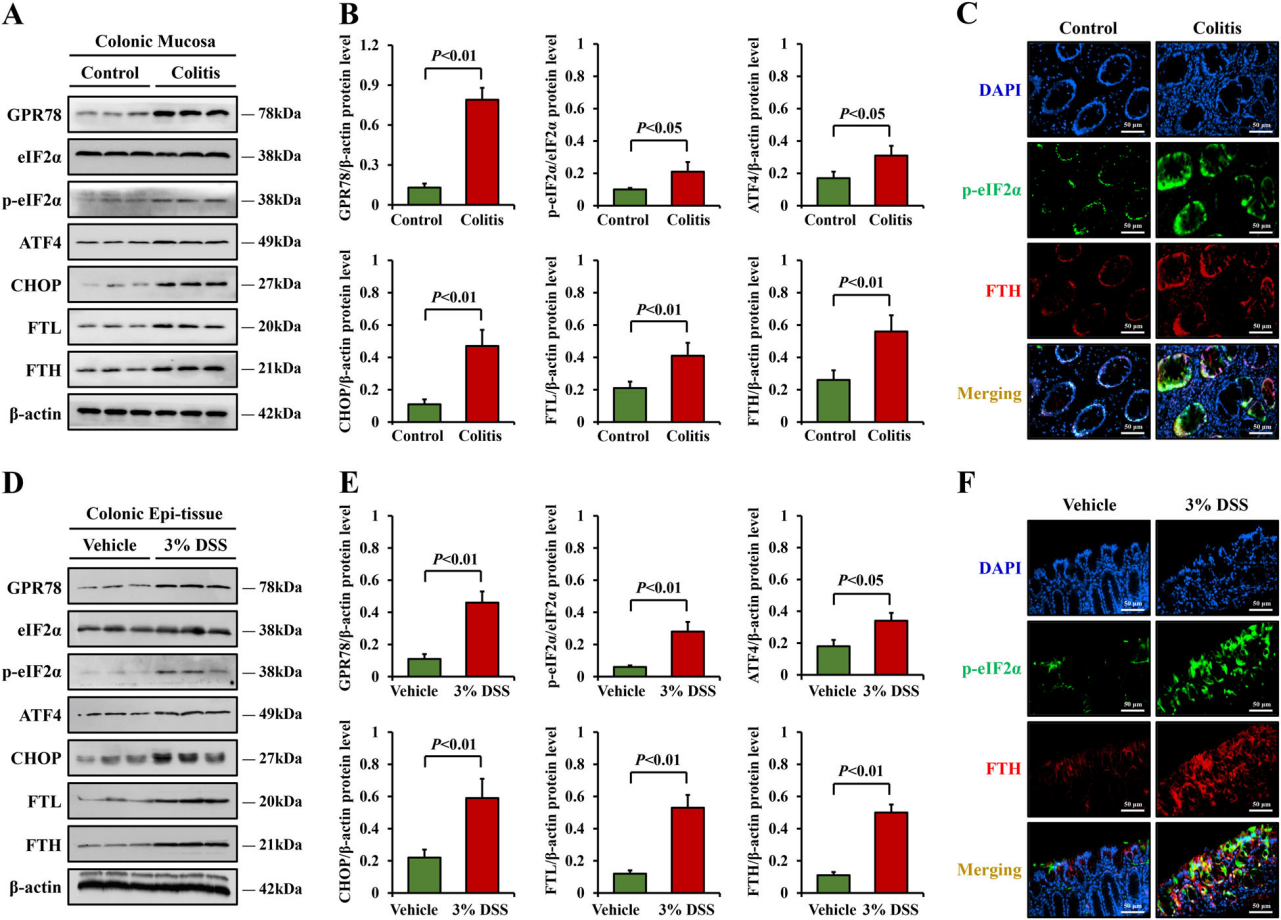
Ferroptosis was mediated by ER stress in colitis. **a**, **b** Western blotting analysis of ER stress-related proteins and FTL, FTH in colonic mucosal tissue from control and UC patients. **c** Double immunofluorescent staining for p-eIF2α and FTH were performed in the human colonic sections. Nuclei was stained with DAPI in blue. Localization of p-eIF2α was stained in green, FTH was visualized in red, and the merging positive signals were visualized in yellow (Scale: 50 μm). **d**, **e** Western blotting analysis of ER stress-related proteins and FTL, FTH in colonic epithelial tissue of DSS-treated mice and vehicle group. β-actin was used as the loading control. **f** Double immunofluorescent staining for p-eIF2α and FTH in colonic sections of mice. Signals were visualized as described in **c** (Scale: 50 μm). Statistical analyses were performed with Student’s *t* tests.

We determined that ferroptosis could be induced in colitis, and it has been reported that ER stress signaling, a vital promotor for UC process, also contributes to the development of ferroptosis^14–16^. In the current study, the levels of proteins involved in the PERK branch of ER stress, including G protein-coupled receptor 78, phosphorylated form of eIF2α (p-eIF2α), ATF4 and CHOP, were elevated in UC samples, a finding in parallel with the increased levels of FTL and FTH (Fig. 4a, b, d, e). We next determined whether ER stress was related to IEC ferroptosis. Indeed, evidence obtained by double immunofluorescent staining for p-eIF2α and FTH revealed that most of the elevated positive signals overlapped in the colonic sections (Fig. 4c, f), suggesting that IEC ferroptosis in colitis might be regulated by ER stress.

**Fig. 5:**
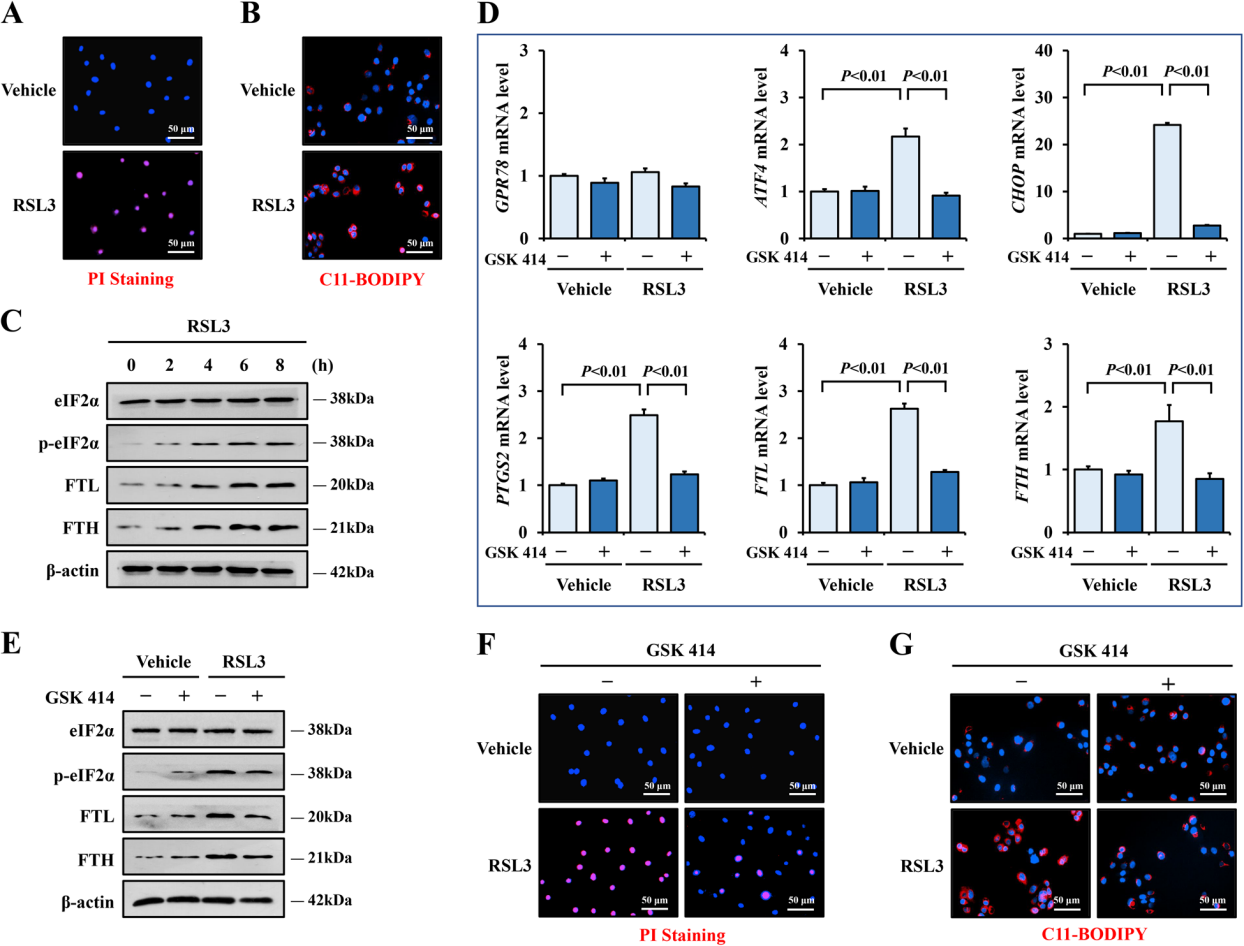
Inhibition of ER stress reduced ferroptosis in intestinal epithelial cells. **a** Cell death of HCoEpiC cells with or without RSL3 (20 μm) challenged for 8 hours was measured by PI staining, positive signal was stained in red (Scale: 50 μm). **b** ROS of HCoEpiC cells with or without RSL3 treatment was detected by C11-BODIPY staining, positive signal was stained in red (Scale: 50 μm). **c** Western blotting analysis of p-eIF2α, eIF2α, FTL, and FTH in cells with RSL3 challenged in the indicated times. β-actin was used as the loading control. **d** Relative mRNA level of GPR78, ATF4, CHOP, PTGS2, FTL, and FTH in HCoEpiC cells were measured by real-time PCR. Cells were treated with or without RSL3 for 8 h, GSK2606414 (GSK 414, 1 μm) was added to the cells 30 mins before RSL3. **e** Western blotting analysis of p-eIF2α, eIF2α, FTL, and FTH in cells with or without RSL3 and GSK 414 treatment. β-actin was used as the loading control. **f** Cell death of indicated cells was tested through PI staining (Scale: 50 μm). **g** ROS of indicated cells was detected by C11-BODIPY staining (Scale: 50 μm). Statistical analyses were performed with Student’s *t* tests (two groups) or one-way ANOVA (more than two groups).

To thoroughly define the relationship between ER stress and ferroptosis, HCoEpiC cells were challenged with RSL3, a canonical inducer of ferroptosis. We observed apparent necrotic cell death and ROS accumulation (Fig. 5a, b) and increased levels of FTL, FTH, and PTGS2 (Fig. 5c, d) in the RSL3-treated cells, which implied that ferroptosis had occurred. In addition, RSL3 administration induced eIF2α phosphorylation and ATF4 and CHOP upregulation (Fig. 5c, d). These data indicated that ER stress signaling was involved in RSL3-induced ferroptosis. GSK 414 is a selective inhibitor of PERK. The application of GSK 414 in vivo and in vitro effectively suppressed the phosphorylation of PERK and prevented the activation of eIF2α/ATF4/CHOP signaling pathway^26,27,35^. When we added GSK 414 to the cells before RSL3 treatment, we were surprised to find that the pretreatment with GSK 414 not only inhibited p-eIF2α, ATF4, and CHOP expression, but also decreased ferroptosis in the RSL3-stimulated cells (Fig. 5d–g). Together, these investigations suggest an important role for ER stress signaling in the mediation of IEC ferroptosis.

### Suppression of ER stress ameliorated colitis via depressing ferroptosis

**Fig. 6:**
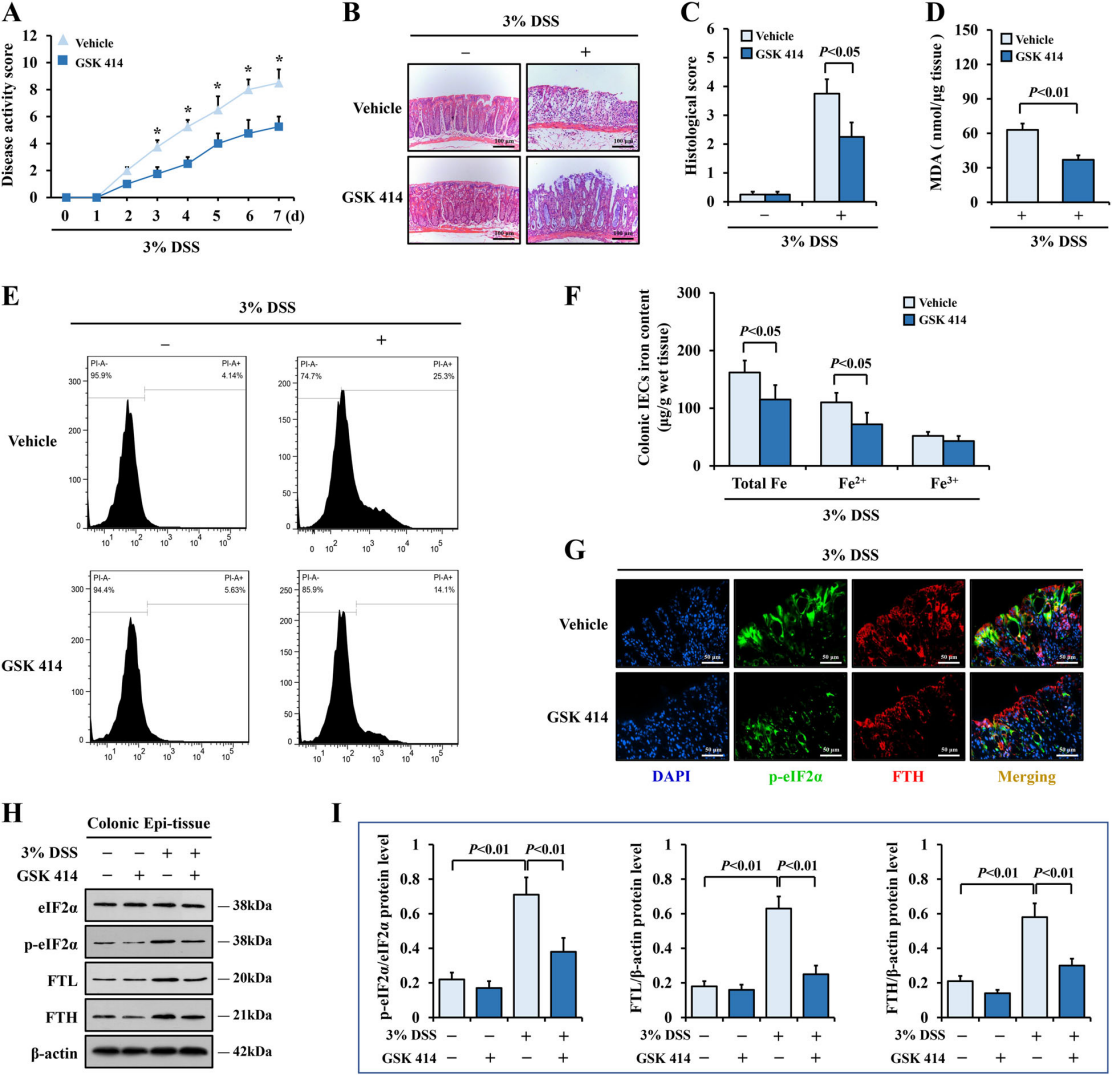
Restraint of ER stress ameliorated colitis via reducing ferroptosis. Mice were treated with GSK2606414 (GSK 414) or vehicle solution. **a** Comparison of the disease activity index between colitis mice with or without GSK 414 challenged. *P < 0.05 versus mice from vehicle group, *n* = 8 in each group. **b** Representative images from H&E staining colonic sections from indicated mice (Scale: 100 μm). **c** Histologic scores were determined according to H&E-stained sections. **d** MDA levels were detected in colonic epithelial tissue from DSS-treated mice with or without GSK 414 challenged. **e** PI-labeled necrotic cell in colonic epithelial cells were analyzed by flow cytometry. **f** Iron levels were measured in colonic epithelial tissue from DSS-treated mice with or without GSK 414 administration. **g** Double immunofluorescent staining for p-eIF2α and FTH in the indicated colonic sections. Nuclei was stained with DAPI in blue. p-eIF2α was stained in green, and FTH was visualized in red. The merging positive signals of p-eIF2α and FTH were visualized in yellow (Scale: 50 μm). **h**, **i** Western blotting analysis of p-eIF2α, eIF2α, FTL, and FTH in colonic epithelial tissue of indicated mice. β-actin was used as the loading control. Statistical analyses were performed with Student’s *t* tests (two groups) or one-way ANOVA (more than two groups).

On the basis of the observations above, we then assessed whether IEC ferroptosis in colitis mice would be altered when ER stress was suppressed in vivo. Interestingly, the application of GSK 414 obviously ameliorated the disease activity of the colitis mice (Fig. 6a) and alleviated the inflammatory injure of the colonic epithelium (Fig. 6b, c). Similar to the results found in vitro, the inhibition of ER stress in colitis mice led to ferroptosis downregulation. As shown in Fig. 6d–f, h, i, the number of necrotic cell death, the MDA and iron contents and the FTL and FTH protein levels in colonic IECs were all decreased in the GSK 414-treated colitis mice. Immunofluorescence detection revealed that the positive signals for p-eIF2α and FTH in the colon sections from GSK 414-challenged colitis mice were decreased simultaneously as further verified by western blotting (Fig. 6g–i), suggesting that ferroptosis was effectively suppressed by blocking ER stress signaling. Collectively, these evidences confirmed that suppressing ER stress signaling inhibited IEC ferroptosis to ameliorate experimental colitis.

### IEC NF-κBp65 exerted protective function against colitis

**Fig. 7:**
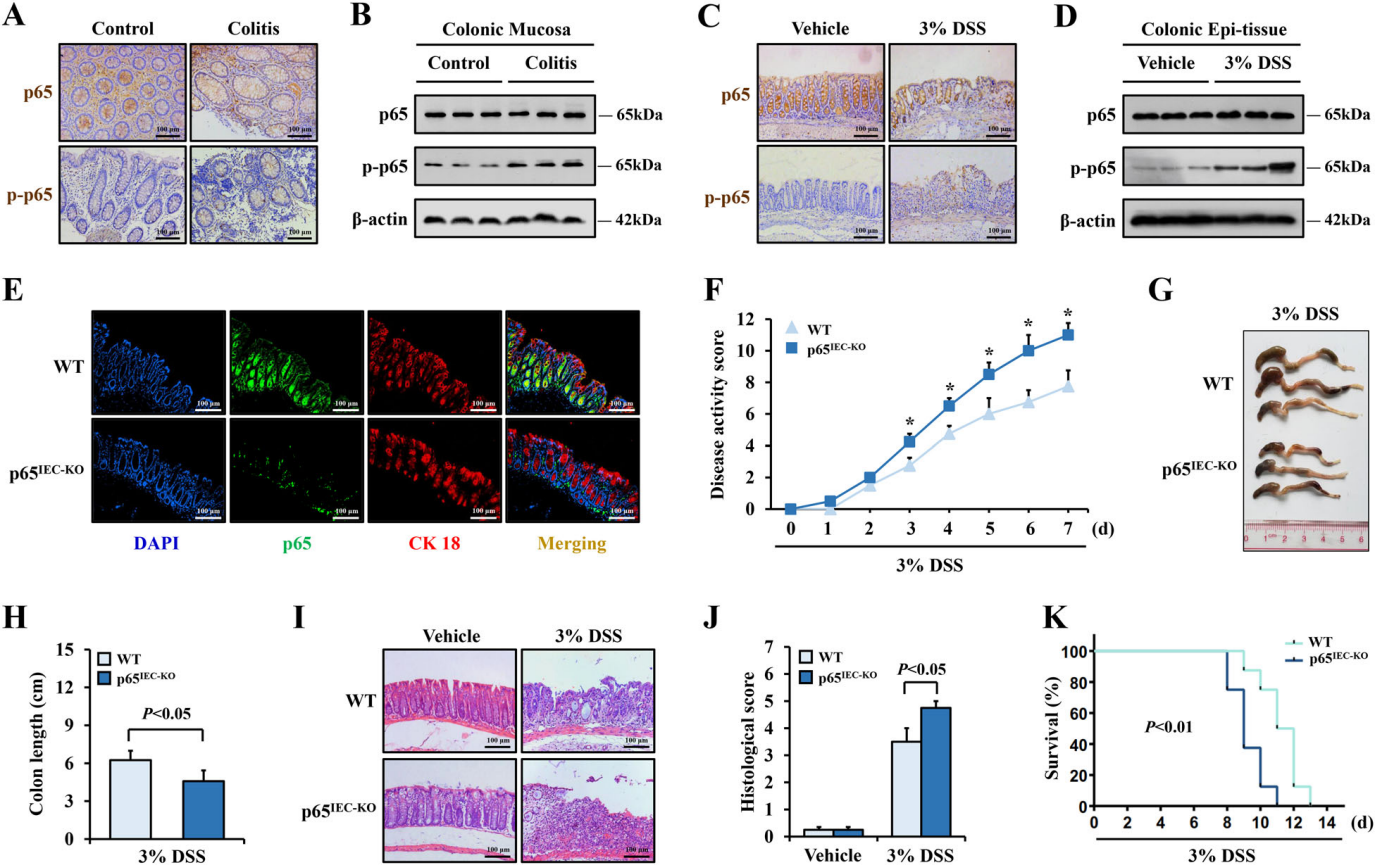
Targeted deletion of intestinal epithelial cellular *NF-κBp65* aggravated DSS-induced colitis in mice. **a** Immunohistochemical staining for p-p65 and p65 in colonic sections from control and UC patients (Scale: 100 μm). **b** Western blotting analysis of p-p65 and p65 in colonic biopsy samples of control and UC patients, β-actin was used as the loading control. **c** Immunohistochemical staining for p-p65 and p65 in colonic sections from DSS-treated mice and vehicle group (Scale: 100 μm). **d** Western blotting analysis of p-p65 and p65 in colonic biopsy samples of DSS-treated mice and vehicle group, β-actin was used as the loading control. **e** Double immunofluorescence staining for p65 and CK 18 in colonic sections of p65^IEC KO^ or WT mice. Nuclei was stained with DAPI in blue. p65 was stained in green and CK 18 was visualized in red, the coincident positive signals were visualized in yellow (Scale: 100 μm). **f** Analysis of the disease activity index of p65^IEC KO^ or WT mice received 3% DSS administration, *n* = 8 in each group. *P < 0.05 versus WT mice. **g**, **h** Comparison of colon length between p65^IEC KO^ or WT mice after DSS treatment for 7 days. **I**, **j** H&E staining colonic sections from indicated mice (Scale: 100 μm), and histologic scores were determined according to H&E-stained sections. **k** Kaplan–Meier survival curve from p65^IEC KO^ or WT mice under DSS treatment, *n* = 8 in each group. Statistical analyses were performed with Student’s *t* tests.

After we confirmed that ER stress signaling contributed to IEC ferroptosis in colitis, we explored the possible regulators of this process. Accordingly, NF-κB and ER stress signaling engage in cross-talk under various pathological and physiological conditions^22^. In the gut epithelium, activated NF-κB exerts protective function against IEC injury and cell death during acute intestinal inflammation. In our study, the immunohistochemistry assay demonstrated that the enhanced p-p65 presented in both the epithelial cells and mesenchymal cells in the colonic sections from UC patients (Fig. 7a). Evidence from the western blot analysis confirmed the elevation of p-p65 in the mucosal tissue samples from the UC patients (Fig. 7b). Similar results were observed in the colonic sections or colonic epithelial tissues of the experimental colitis mice (Fig. 7c, d).

To clarify the function of IEC NF-κBp65 in colitis, we ablated the p65 gene specially in mouse IECs (p65^IEC-KO^). As measured by double immunofluorescence assay, the signal of p65 vanished in the intestinal epithelium from the p65^IEC-KO^ mice (Fig. 7e). After DSS challenge, the p65^IEC-KO^ mice presented higher disease activity and shorter colon lengths on day 7 than were presented by WT mice (Fig. 7f–h). Furthermore, histopathologic analysis revealed that the deficiency of NF-κBp65 in the IECs led to more severe epithelial destruction and intestinal inflammation in colitis mice (Fig. 7i, j). Finally, p65^IEC-KO^ mice had remarkably fewer survival days than their WT littermates when consecutive treated with DSS (Fig. 7k). These data collectively implied that the activated NF-κBp65 in IECs had protective function against DSS-induced inflammation-related injury.

### Deficiency of IEC NF-κBp65 led to upregulated ER stress-mediated ferroptosis in colitis

**Fig. 8:**
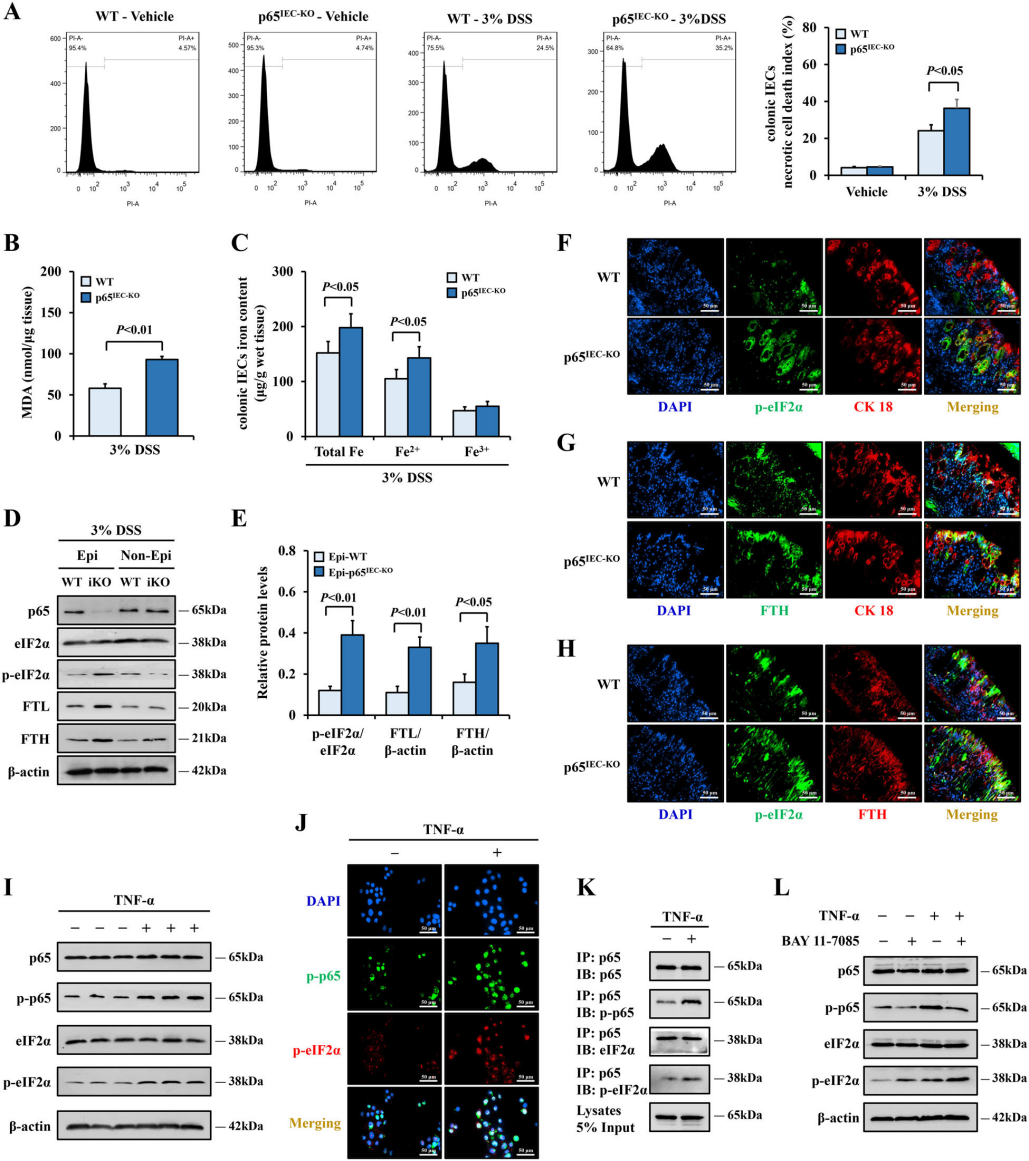
Phosphorylation of intestinal epithelial NF-κBp65 inhibited ER stress-mediated ferroptosis in colitis via interacting with eIF2α. **a** Necrotic cell death in colonic epithelial tissues from p65^IEC-KO^ or WT mice was labeled by PI and analyzed through flow cytometry. **b**, **c** MDA levels and iron levels were detected in colonic epithelial tissues from p65^IEC-KO^ or WT mice received DSS administration. **d**, **e** Western blotting analysis of p65, p-eIF2α, eIF2α, FTL, and FTH in colonic epithelial/non-epithelial tissues from p65^IEC KO^ or WT mice after DSS treatment. β-actin was used as the loading control. **f** Double immunofluorescence staining for p-eIF2α and CK 18 in colonic sections of p65^IEC KO^ or WT mice with DSS challenged. Nuclei was stained with DAPI in blue. p-eIF2α was stained in green and CK 18 was visualized in red, the coincident positive signals were visualized in yellow (Scale: 50 μm). **g** Double immunofluorescence staining for FTH and CK 18 in colonic sections. FTH was stained in green, and CK 18 was visualized in red, the merging positive signals were visualized in yellow (Scale: 50 μm). **h** Double immunofluorescence staining for p-eIF2α and FTH in colonic sections. p-eIF2α was stained in green and FTH was visualized in red, the coincident positive signals were visualized in yellow (Scale: 50 μm). **i** Western blotting of p-p65, p65 p-eIF2α, and eIF2α in HCoEpiC cells with or without TNF-α treatment (40 ng/ml, 1 h). β-actin was used as the loading control. **j** Immunofluorescence staining for p-p65 and p-eIF2α in HCoEpiC cells. Nuclei was stained with DAPI in blue. p-p65 was stained in green and p-eIF2α was visualized in red, the overlapped positive signals were visualized in yellow (Scale: 50 μm). **k** HCoEpiC cells with or without TNF-α challenged were subjected to immunoprecipitation with an anti-p65 antibody. Co-immunoprecipitated endogenous p-p65, eIF2α, and p-eIF2α were detected with corresponding antibodies. *IP* immunoprecipitate, *IB* immunoblot. **l** Analysis of the effect of BAY 11-7085 (10 μm, adding to the indicated wells 15 mins before TNF-α administration) on the activation of p65 and eIF2α in cells with or without TNF-α treatment through western blotting. β-actin was used as the loading control. Statistical analyses were performed with Student’s *t* tests (two groups) or one-way ANOVA (more than two groups).

Given that activated NF-κBp65 protected IECs from inflammatory injury in colitis and that NF-κB is thought to be involved in the regulation of ER stress and ferroptosis process, we conjectured that NF-κBp65 might regulate ER stress-mediated ferroptosis. As expected, DSS-treated p65^IEC-KO^ mice displayed a markedly higher percentage of necrotic cell death in colonic IECs (35.2% in DSS-treated p65^IEC-KO^ mice vs 24.5% in DSS-treated WT mice) and higher levels of MDA and iron compared with that in the WT mice (Fig. 8a–c). Interestingly, the increased necrotic cell death, MDA, and iron levels in DSS-challenged p65^IEC-KO^ mice could be thoroughly rescued by Fer1 treatment (Supplementary figure 2). These data suggested a suppressive function of IEC-derived NF-κBp65 on ferroptosis. As ferroptosis of the IECs was mediated by ER stress, we proposed that NF-κBp65 rescued ferroptosis by inhibiting ER stress signaling. Indeed, p65^IEC-KO^ mice had increased levels of p-eIF2α and ferritin proteins after DSS administration (Fig. 8d, e), and the double immunofluorescence further showed that the p-eIF2α and FTH signatures were increased synchronously (Fig. 8f–h), indicating a very promising function of NF-κBp65 in suppressing ER stress/ferroptosis signaling in IECs. In brief, these data confirmed that NF-κBp65 deficiency in the IECs exacerbated ER stress-mediated ferroptosis in experimental colitis.

### Phosphorylation of NF-κBp65 inhibited ER stress by interacting with eIF2α

Next, we sought to determine how NF-κBp65 regulated ER stress signaling. We treated HCoEpiC cells with TNF-α, a canonical activator of NF-κB. The TNF-α administration increased the levels of p-p65, and simultaneously induced the phosphorylation of eIF2α (Fig. 8i). Immunofluorescent staining showed few positive p-p65 signals and negligible levels of p-eIF2α in the cells under physiological conditions, but abundant and overlapping positive signals of p-p65 and p-eIF2α were observed in the cells treated with TNF-α (Fig. 8j). To confirm the mechanism of the p65 and eIF2α interaction, we conducted a coimmunoprecipitation experiment. The results indicated that p65 interacted with eIF2α by binding to it. (Fig. 8k). Surprisingly, we observed that inhibiting the activation of NF-κBp65 by BAY 11-7085 led to enhanced expression of p-eIF2α under both normal conditions and TNF-α challenge (Fig. 8l), indicating that phosphorylation of p65 exerted a suppressive function on eIF2α activation. These results demonstrated the critical role of activated NF-κBp65 in inhibiting ER stress signaling through its interaction with eIF2α.

## Discussion

UC is an inflammatory-associated disease characterized by persistent damage to epithelial cells of the colon. So far, the mechanism of UC pathogenesis has not been completely understood. Herein, we first proposed that iron- and ROS-dependent ferroptosis involves in colonic epithelial cell death of UC. More importantly, we revealed that IEC ferroptosis in UC was mediated via ER stress signaling and primarily suppressed by phosphorylated-NF-kBp65.

In recent decades, existing studies have reported that iron and ROS contribute to UC development^9,10^. However, the detailed injured mechanism of iron and ROS has not been well described. Ferroptosis is a novel form of regulated necrosis that triggered by excess ferrous iron and accumulated ROS^3^. Our data showed that several ferroptosis-associated gene transcripts notably altered in UC specimens. Shrunken mitochondria, increased necrotic cell death, and upregulated iron and ROS in colonic mucosa/IECs were observed in human UC and DSS-induced murine colitis. These data suggested that ferroptosis was a cause of colitis-associated IEC cell death as was further verified through the administration of Fer1, a specific inhibitor of ferroptosis, to the mice.

ER stress is well described as a significant modulator of inflammatory damage and even triggers cell death if it persistent exists^36^. Genetic abnormalities and aberrant environmental factors of the gut account for the induction of ER stress in IECs of UC. A genetic study revealed primary aberrance in several ER homeostasis-associated genes, including *AGR2*, *XBP1,* and *ORMDL3* in UC patients^37^. The exposure to high levels of inflammatory cytokines such as TNF-α disturbs protein folding function of ER and alters ER homeostasis in IECs^38,39^. Moreover, various microbiota and microbial metabolites in the colonic lumen of UC activate immunity response and further trigger IEC ER stress^40^. Our previous study demonstrated that ER stress-mediated colonic IEC apoptosis in UC^17^. The current data showed that eIF2α/ATF4/CHOP signaling was upregulated in UC-derived colonic IECs. Interestingly, we also determined that ER stress participated in the development of ferroptosis because signatures of ER stress and ferroptosis overlapped mainly in the colonic IECs of UC. ER stress-facilitated ferroptosis has been implicated in previous researches. Park, et al.^14^ found that whole cigarette smoke condensates induce ferroptosis in bronchial epithelial cells via ER stress. To verify this mechanism, we administered GSK 414, a specific inhibitor of the PERK branch of ER stress signaling both in vivo and in vitro. It came out that GSK 414 treatment clearly downregulated IEC necrotic cell death, iron contents, and ROS in the mice with colitis. Moreover, in ferroptosis inducer RSL3-challenged HCoEpiC cells, pretreatment with GSK 414 notably rescued cells from ferroptosis. These data are consistent with the results from the studies described above and indicate a new role for ER stress, showing that it contributes not only to apoptosis but also to ferroptosis in the IECs of UC. Nevertheless, our results did not clarify the concrete regulatory mechanism of ER stress on ferroptosis. Therefore, further studies are needed.

Interestingly, accumulating evidence indicates a protective role of NF-κB in the IECs, promoting resistance to damage and cell death. Our previous study revealed that blocking the activity of NF-κB with BAY 11-7085 in vivo aggravated IECs apoptosis in UC^20^. Other studies have found that mice with gene deletion of NF-κB member were more susceptible to inflammatory damage and death^41,42^. In addition, NF-κB is a critical regulator for both ER stress signaling and ferroptosis process^21,22^. Consistent with previous literature, the current data showed that mice lacking NF-κBp65 in IECs led to more serious DSS-induced colitis. Interestingly, our study also provides exquisite evidence that the deletion of IEC NF-κBp65 in mice caused upregulated IEC necrotic cell death and increased levels of ferrous iron and ROS in colitis, implicating that NF-κBp65 resisted IEC ferroptosis in colitis. Noteworthily, our further results elucidated that NF-κBp65 suppressed ferroptosis via downregulation of ER stress, as DSS-treated p65^IEC-KO^ mice displayed synchronous elevation of p-eIF2α and FTH in colonic epithelial cells. These observations provide a new perspective for recognizing IEC NF-κBp65 against ER stress-mediated ferroptosis in UC.

We further clarified how NF-κBp65 regulates ER stress-associated ferroptosis. Through coimmunoprecipitation in HCoEpiC cells, NF-κBp65 was found to inhibit ER stress-mediated ferroptosis via direct interaction with eIF2α, a key component of the PERK branch of ER stress. The application of BAY 11-7085 in cells confirmed that activated NF-κBp65 exerted a suppressive function on the phosphorylation of eIF2α. This result was different from that of previous reports, indicating that NF-κB can be activated by ER stress signaling and that eIF2α acts as a direct upstream mediator for NF-κB activation^20,43–45^. Herein, ER stress inducer thapsigargin- (4 μm) and tunicamycin- (10 μg/ml) treated HCoEpiC cell presented enhanced level of p-p65, which could be blocked by GSK 414 (data not shown). These results are consistent with that from previous literature^43,44^. However, when we pretreated the cells with BAY 11-7085 to inhibit the activity of p65 before thapsigargin/tunicamycin administration, the phosphorylation of eIF2α was enhanced (data not shown), a finding that was similar to the data shown in Fig. 8l. These observations indicated a novel relationship between ER stress and NF-κB, suggesting a feedback loop between them. Generally speaking, the induction of ER stress can activate NF-κBp65, and phosphorylated-NF-κBp65 produces negative feedback on ER stress and subsequently suppresses ER stress-mediated ferroptosis.

Conclusively, our findings revealed that ferroptosis contributes to IEC cell death in UC and that ER stress signaling mediates IEC ferroptosis. Phosphorylated-NF-κBp65 suppresses ER stress-mediated IEC ferroptosis by interacting with eIF2α. These data suggest that inhibiting ferroptosis will be a new therapeutic method and that the phosphorylation of NF-κBp65 is a potential therapeutic target for UC.

## Supplementary information

Supplementary figure 1

Supplementary figure 2

Supplementary table 1

Supplementary table 2

Supplementary table 3

Supplementary figure legends

Supplementary materials and methods

Author contribution

Reproducibility checklist
