# Supplementary figures and images for: Ferroptosis involves in intestinal epithelial cell death in ulcerative colitis
Source: Cell Death Dis. 2020 Feb 3;11(2):86. doi: 10.1038/s41419-020-2299-1 (PMC6997394; doi:10.1038/s41419-020-2299-1)

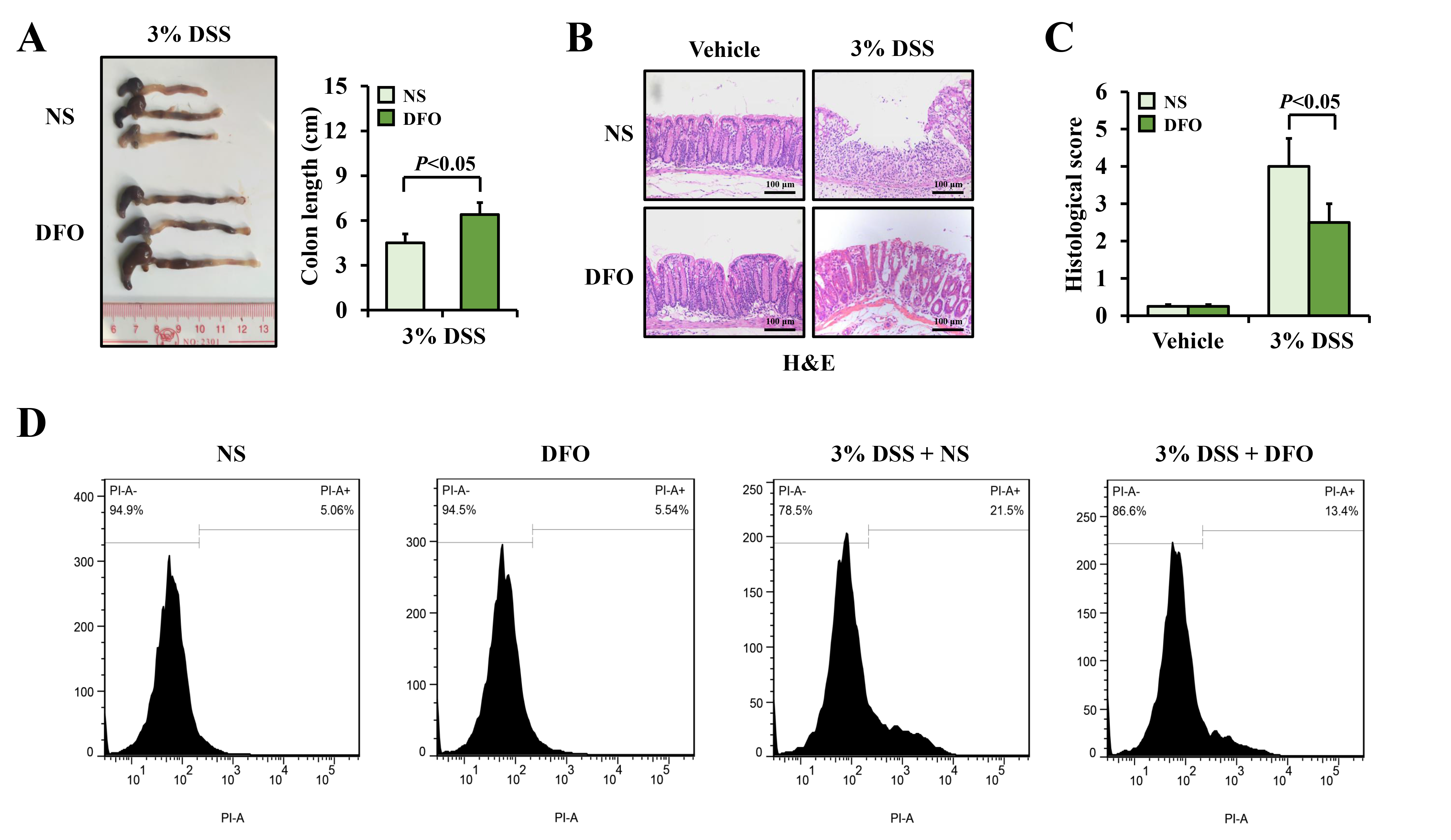

Supplement: Supplementary file 1 — Supplementary figure 1 [file 41419_2020_2299_MOESM1_ESM.tif]

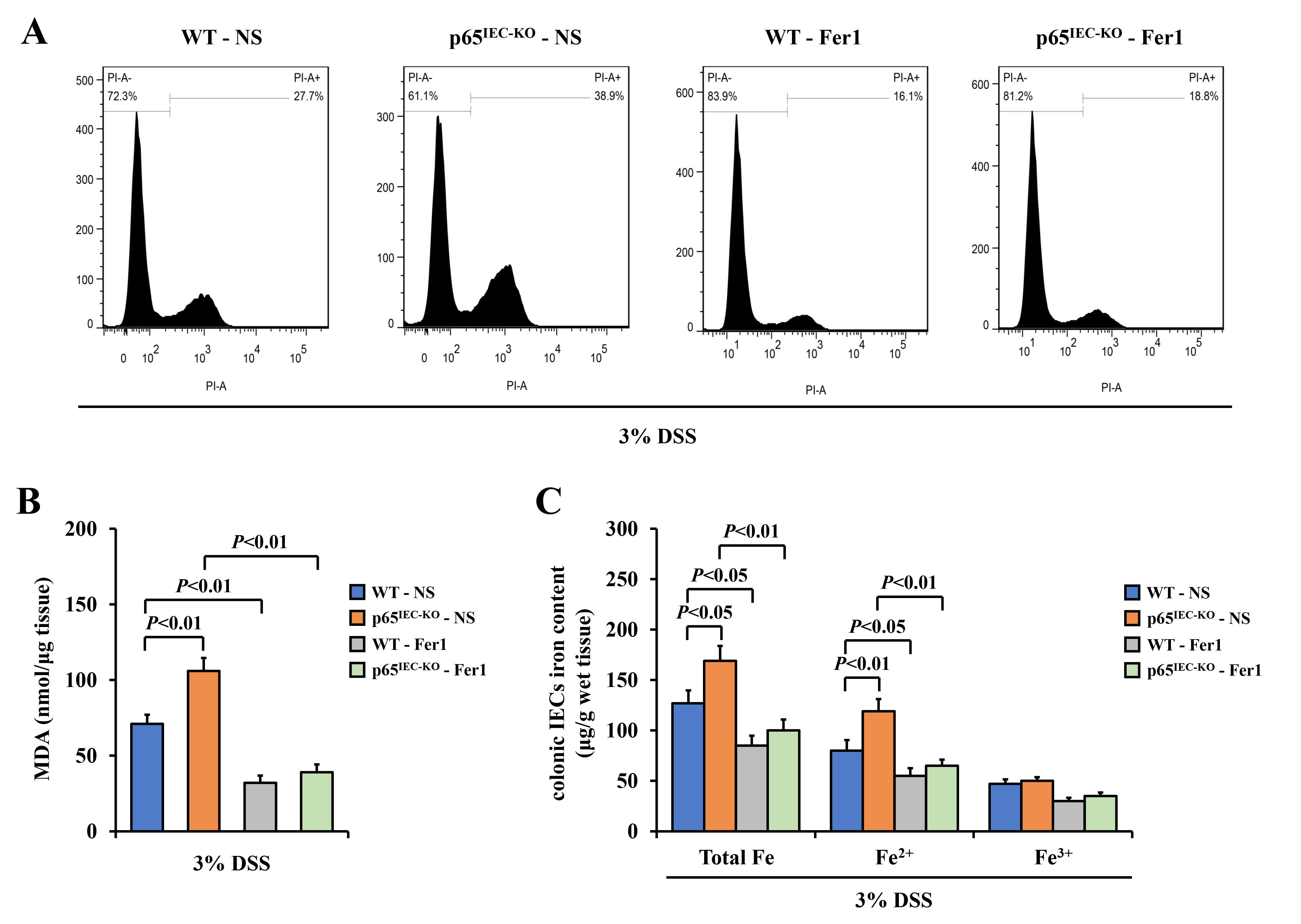

Supplement: Supplementary file 2 — Supplementary figure 2 [file 41419_2020_2299_MOESM2_ESM.tif]
