# Supplementary material for: Ferroptosis involves in intestinal epithelial cell death in ulcerative colitis
Source: Cell Death Dis. 2020 Feb 3;11(2):86. doi: 10.1038/s41419-020-2299-1 (PMC6997394; doi:10.1038/s41419-020-2299-1)
Supplement: Supplementary file 3 — Supplementary table 1 [file 41419_2020_2299_MOESM3_ESM.docx]

**Supplementary table 1**

| **Gene** | **Regulation** | **Fold Change** | ***P*** |
| --- | --- | --- | --- |
| *CXCL1* | up | 54.1169 | 0.0023 |
| *NOS2* | up | 31.4031 | 0.0036 |
| *CXCL3* | up | 13.1421 | 0.0045 |
| *CXCL2* | up | 10.0078 | 0.0190 |
| *IL1A* | up | 7.3509 | 0.0384 |
| *IFNG* | up | 4.5529 | 0.0267 |
| *TAP2* | up | 5.9036 | 0.0391 |
| *IRS1* | up | 2.1461 | 0.0485 |
| *LAMC2* | up | 3.2648 | 0.0263 |
| *OSMR* | up | 2.3788 | 0.0121 |
| *PML* | up | 2.8758 | 0.0045 |
| *STAT1* | up | 3.1537 | 0.0395 |
| *BIRC3* | up | 4.1708 | 0.0161 |
| *GNA15* | up | 4.3422 | 0.0114 |
| *HIF1A* | up | 3.3845 | 0.0077 |
| *TAP1* | up | 3.4651 | 0.0176 |
| *F2R* | up | 3.8542 | 0.0246 |
| *COL6A3* | up | 3.7911 | 0.0034 |
| *CASP1* | up | 4.1611 | 0.0007 |
| *WNT5A* | up | 3.6333 | 0.0070 |
| *FLT1* | up | 2.2280 | 0.0083 |
| *KDR* | up | 1.9837 | 0.0271 |
| *KIT* | up | 2.8361 | 0.0001 |
| *VWF* | up | 2.6478 | 0.0088 |
| *CD44* | up | 1.7891 | 0.0409 |
| *HLA-B* | up | 2.1889 | 0.0149 |
| *HLA-F* | up | 2.5925 | 0.0161 |
| *SOD2* | up | 2.4436 | 0.0130 |
| *HLA-G* | up | 2.0567 | 0.0103 |
| *CREB3L2* | up | 1.9468 | 0.0012 |
| *DOCK4* | up | 1.5464 | 0.0220 |
| *HLA-C* | up | 1.7106 | 0.0264 |
| *IFNAR2* | up | 1.6349 | 0.0477 |
| *HOMER1* | up | 1.5732 | 0.0453 |
| *ITGAM* | up | 1.4736 | 0.0300 |
| *BCL6* | up | 1.8345 | 0.0038 |
| *HSPG2* | up | 1.6724 | 0.0056 |
| *HSP90B1* | up | 1.5523 | 0.0057 |
| *PLCB1* | up | 1.5702 | 0.0213 |
| *KSR1* | up | 1.7185 | 0.0067 |
| *STAT3* | up | 1.7238 | 0.0016 |
| *CAV2* | up | 1.1728 | 0.0453 |
| *MAPK11* | up | 1.2909 | 0.0210 |
