# Supplementary material for: Ferroptosis involves in intestinal epithelial cell death in ulcerative colitis
Source: Cell Death Dis. 2020 Feb 3;11(2):86. doi: 10.1038/s41419-020-2299-1 (PMC6997394; doi:10.1038/s41419-020-2299-1)
Supplement: Supplementary file 4 — Supplementary table 2 [file 41419_2020_2299_MOESM4_ESM.docx]

**Supplementary table 2**

| **Antibody** | **Company** | **Applications** | **Dilution** |
| --- | --- | --- | --- |
| **MPO** | Merck (Darmstadt, Germany) | IHC | 1:200 |
| **FTH** | Abcam (Cambridge, MA, USA) | IHC, IF, WB | 1:200 in IHC & IF  1:2000 in WB |
| **p65** | Merck (Darmstadt, Germany) | IHC, IF | 1:300 |
| **p-p65** | Merck (Darmstadt, Germany) | IHC | 1:300 |
| **CK 18** | Abcam (Cambridge, MA, USA) | IF | 1:300 |
| **p-eIF2α** | Cell Signaling Technology (Danvers, MA, USA) | IF, WB | 1:200 in IF  1:1500 in WB |
| **FTL** | Abcam (Cambridge, MA, USA) | WB | 1:2000 |
| **eIF2α** | Cell Signaling Technology (Danvers, MA, USA) | WB | 1:3000 |
| **ATF4** | Cell Signaling Technology, (Danvers, MA, USA) | WB | 1:2000 |
| **CHOP** | Cell Signaling Technology (Danvers, MA, USA) | WB | 1:2000 |
| **p65** | Cell Signaling Technology (Danvers, MA, USA) | WB | 1:3000 |
| **p-p65** | Cell Signaling Technology (Danvers, MA, USA) | WB | 1:3000 |
| **GPR78** | Merck (Darmstadt, Germany) | WB | 1:3000 |
| **β-actin** | Merck (Darmstadt, Germany) | WB | 1:3000 |

IHC: Immunohistochemical staining;

IF: Immunofluorescence staining;

WB: Western Blotting.
