# Supplementary material for: Ferroptosis involves in intestinal epithelial cell death in ulcerative colitis
Source: Cell Death Dis. 2020 Feb 3;11(2):86. doi: 10.1038/s41419-020-2299-1 (PMC6997394; doi:10.1038/s41419-020-2299-1)
Supplement: Supplementary file 5 — Supplementary table 3 [file 41419_2020_2299_MOESM5_ESM.docx]

**Supplementary table 3**

| **Gene** | **Species** | **Primer** | **Sequence** |
| --- | --- | --- | --- |
| ***PTGS2*** | Mouse | Forward primer  Reverse primer | 5’TGAGCAACTATTCCAAACCAGC3’  5’ CACGTAGTCTTCGATCACTATC3’ |
| ***GPX4*** | Mouse | Forward primer  Reverse primer | 5’ GCCTGGATAAGTACAGGGGTT 3’  5’ CATGCAGATCGACTAGCTGAG 3’ |
| ***β-actin*** | Mouse | Forward primer  Reverse primer | 5’ GGCTGTATTCCCCTCCATCG 3’  5’ CCAGTTGGTAACAATGCCATGT 3’ |
| ***GPR78*** | Human | Forward primer  Reverse primer | 5’GAGACAGTGGGTAGGGAAGTGC3’  5’GAACAAATCGGAACAATGCTAA 3’ |
| ***ATF4*** | Human | Forward primer  Reverse primer | 5’ CCTCGATTCCAGCAAAGCA 3’  5’ CCATCCACAGCCAGCCATT 3’ |
| ***CHOP*** | Human | Forward primer  Reverse primer | 5’ CCACTCTTGACCCTGCTTC 3’  5’ CCACTCTGTTTCCGTTTCC 3’ |
| ***PTGS2*** | Human | Forward primer  Reverse primer | 5’ CTTGGGTGTCAAAGGTAAA 3’  5’ ACTGATGCGTGAAGTGCTG 3’ |
| ***FTL*** | Human | Forward primer  Reverse primer | 5’ CTTGGGTGTCAAAGGTAAA 3’  5’ ACTGATGCGTGAAGTGCTG 3’ |
| ***FTH*** | Human | Forward primer  Reverse primer | 5’ ATCTGGCTTGGCGGAATAT 3’  5’ TCAAAGACAACACCTGGGTA 3’ |
| ***β-actin*** | Human | Forward primer  Reverse primer | 5’ GTCTTCCCCTCCATCGTG 3’  5’ AGGGTGAGGATGCCTCTCTT 3’ |
