# Supplementary material for: Ferroptosis involves in intestinal epithelial cell death in ulcerative colitis
Source: Cell Death Dis. 2020 Feb 3;11(2):86. doi: 10.1038/s41419-020-2299-1 (PMC6997394; doi:10.1038/s41419-020-2299-1)
Supplement: Supplementary file 6 — Supplementary figure legends [file 41419_2020_2299_MOESM6_ESM.docx]

**Supplementary figure legends**

**Supplementary Figure 1 Deferoxamine treatment reduced ferroptosis to ameliorate experimental colitis.**

Mice were treated with deferoxamine (DFO) or normal saline (NS). **(A)** Comparison of colon length between colitis mice received DFO or NS treatment. **(B)** Representative images of H&E staining colonic sections from DFO group and NS group mice (Scale: 100 μm). (**C**) Histologic scores were determined according to H&E-stained sections. **(D)** PI-positive cells were detected through flow cytometry to analyse necrotic cell death in colonic epithelial tissues of mice.

**Supplementary Figure 2 Ferrostatin-1 treatment rescued the upregulated ferroptosis in DSS-challenged mice with deficiency of intestinal epithelial cellular NF-κBp65.**

DSS-challenged p65^IEC-KO^ or WT mice were treated with ferrostatin 1 (Fer1) or normal saline (NS). **(A)** Necrotic cell death in colonic epithelial tissues from indicated p65^IEC-KO^ or WT mice was labeled by PI and analysed through flow cytometry. **(B and C)** MDA and iron levels were detected in colonic epithelial tissues from indicated p65^IEC-KO^ or WT mice.

**Supplementary table 1 Ulcerative colitis related gene expressions analysis in colonic biopsy samples from control and ulcerative colitis patients.**

**Supplementary table 2 Antibodies involved in this study.**

**Supplementary table 3 Primers used for RT-PCR analysis.**
