# Supplementary material for: Ferroptosis involves in intestinal epithelial cell death in ulcerative colitis
Source: Cell Death Dis. 2020 Feb 3;11(2):86. doi: 10.1038/s41419-020-2299-1 (PMC6997394; doi:10.1038/s41419-020-2299-1)
Supplement: Supplementary file 7 — Supplementary materials and methods [file 41419_2020_2299_MOESM7_ESM.docx]

**Supplementary materials and methods**

**Determination of the Disease Activity Index**

Over the experiment, mice were observed and recorded every day to determine the disease activity index score on a scale of 1-4 as the sum of weight loss, stool consistency and fecal occult blood (Hemoccult fecal occult blood test, Beckman Coulter, Fullerton, CA), the detailed criteria were described previously [1].

**Histologic Analysis**

The colonic sections were subjected to hematoxylin and eosin (H&E) staining for histological analysis. Histological scores were assessed blindly basis on the degree of inflammation as previously described [1].

**Isolation of colonic epithelial cells**

This protocol of isolation of murine colonic epithelial cells was performed according to other study [2] with some modifications. Briefly, the whole colon of mice was cut into 2-3 mm lengths and subjected to 10ml digestive solution (300U/ml collagenase Ⅺ (Merck), 0.1mg/ml dispase (Merck), in HBSS), followed shaking vigorously at 37℃ for 30 minutes. Next, tissue/ digestive solution was centrifuged at 1200 revs/min for 3 minutes and the supernatant was discarded. The precipitate was resuspended with 10ml dispersive solution (5% fetal bovine serum, 2% sorbitol, in DMEM medium) and vigorously pipetted 150 times and let rest for 1minute, then removed the supernatant carefully to a new 50ml tube, added new dispersive solution into the precipitates, repeated this whole step three times. Centrifuged the collected supernatant at 300 revs/min for 3 minutes, discarded the supernatant and kept the precipitates for further utilization.

**Immunohistochemical and** **immunofluorescent staining**

For immunohistochemical staining, colon sections were incubated with 3% hydrogen to block endogenous peroxidase activity. Antigen was unmasked in boiling EDTA buffer (pH 8.0) for 3 minutes. After blocked with 10% goat serum (Merck), sections were incubated with corresponding primary antibody. For double immunofluorescence staining, after antigen retrieval, sections were incubated with antibody of the first protein overnight at 4 ℃, followed by the related biotin-conjugated secondary antibody and streptavidin Alexa 488 or 594 (Molecular Probes, Beijing, China). After completing the first protein staining, the sections were then incubated and detected the secondary protein. The nuclei were stained with 4′6-diamidino-2-phenylindole dihydrochloride (DAPI, Invitrogen, Carlsbad, CA, USA). The images were assessed in a blinded fashion. The involved antibodies were provided in Supplementary table 2.

**Western blotting**

Proteins extracted from colonic mucosa/epithelia or cell lysates were subjected to SDS-PAGE and electrotransfered to nitrocellulose membranes. Nonspecific binding sites were blocked in 5% skim milk at room temperature for 2 hours. Membranes were then probed with primary antibodies at 4 ℃ overnight, then incubated with the appropriate peroxidase-conjugated secondary antibody. The signal was visualized by ECL chemiluminescence (Thermo Fisher Scientific, Waltham, MA, USA). Densitometric analysis of Western blots was performed using NIH Image software. The involved antibodies above were provided in Supplementary table 2.

**Quantitative Real-time PCR**

For quantitative real-time PCR (qPCR), total RNA was extracted from colonic mucosal/epithelial tissues or cells, then cDNA synthesized was performed as the manufacturer’s instructions described (Invitrogen). The mRNA levels of FTL, FTH, PTGS2, GPR78, ATF4 and CHOP, were analyzed by a Mini Opticon Real-time PCR System (BioRad, Hercules, CA, USA) with SYBR Green (Invitrogen). Results were normalized to β-actin. Primer sequences of above genes were provided in Supplementary table 3.

**Measurement of iron contents**

The iron contents were measured using an iron assay kit (Abcam, Cambridge, MA, USA) as the manufacturer’s instructions described.

**Assessment of lipid peroxidant**

Malondialdehyde (MDA) contents of the colonic mucosal/epithelial tissues were detected using a lipid peroxidation assay kit (Abcam) following standard instructions. The level of lipid ROS in the cells was tested through C11-BODIPY (581/591) (Invitrogen) staining. HCoEpiC cells were placed into cell slides on 12-well plates. After drug treatment, cells were washed twice with phosphate buffer saline (PBS) and incubated with 10 μM C11-BODIPY for 20 minutes. Then, the cells were washed twice with PBS. After staining the nuclei with DAPI, the slides were observed with a fluorescent microscope.

**Necrotic cell death measurement**

For the detection of necrotic cells in the colon, propidium iodide (PI, Merck) was dissolved in phosphate-buffered saline (PBS) to final concentration of 100 μg/mL and injected to mice intravenously (200 ul per mouse) via the tail vein 4 hours before sacrificed. Then colons were harvested and epithelial cells were isolated. PI-positive epithelial cells were detected immediately by flow cytometry. For HcoEpiC cells, necrotic cell death was assessed through a propidium iodide (PI) staining kit (KeyGEN BioTECH, Nanjing, China) according to the manufacturer’s instructions.

**Co-immunoprecipitation**

HCoEpiC cells pretreated as indicated were collected and placed into 1.5 mL tubes with 500 ul Cell Lysis Buffer (Thermo Fisher Scientific) and 10 ug antibody of rabbit anti-p65 (Cell Signaling Technology, Danvers, MA, USA) at 4 ℃ overnight with mixing. The cell lysis/antibody mixture was poured to a new 1.5 mL tube containing pre-washed Protein A/G Magnetic Beads (Thermo Fisher Scientific) and incubated at room temperature for 1 hour with mixing. The flow-through was removed, the magnetic beads were washed twice with tris-buffered saline contained 5% Tween-20 (TBST). Then, 100 μl elution buffer (0.1M glycine, pH2.0) was added to the beads and gently mixed for 10 minutes. Finally, we collected the fluids and performed further western blotting analysis.

**Transmission electron microscopy**

Fresh colonic tissues were fixed with ice-cold 2.5% glutaral. Transmission electron microscopy was performed using a transmission electron microscope (JEOL, Tokyo, Japan) at the electron microscopy core lab of Sun Yat-Sen University.

**Reference**

1. Zeng LX, Tao J, Liu HL, Tan SW, Yang YD, Peng XJ, et al. β-Arrestin2 encourages inflammation-induced epithelial apoptosis through ER stress/PUMA in colitis. *Mucosal Immunol.* **8**, 683-695 (2015).
2. Evans GS, Flint N, Somers AS, Eyden B, Potten CS. The development of a method for the preparation of rat intestinal epithelial cell primary cultures. *J Cell Sci.* **101**, 219-231 (1992).
